# Optimising Surface Roughness and Density in Titanium Fabrication via Laser Powder Bed Fusion

**DOI:** 10.3390/mi14081642

**Published:** 2023-08-20

**Authors:** Hany Hassanin, Mahmoud Ahmed El-Sayed, Mahmoud Ahmadein, Naser A. Alsaleh, Sabbah Ataya, Mohamed M. Z. Ahmed, Khamis Essa

**Affiliations:** 1 School of Engineering, Technology, and Design, Canterbury Christ Church University, Canterbury CT1 1QU, UK; 2 Department of Industrial and Management Engineering, Arab Academy for Science, Technology and Maritime Transport, Alexandria 21599, Egypt; dr.mahmoudelsayed12@gmail.com; 3 Department of Production Engineering and Mechanical Design, Tanta University, Tanta 31512, Egypt; m.ahmadein@f-eng.tanta.edu.eg; 4 Department of Mechanical Engineering, Imam Mohammad Ibn Saud Islamic University (IMSIU), Riyadh 11432, Saudi Arabia; naalsaleh@imamu.edu.sa (N.A.A.); smataya@imamu.edu.sa (S.A.); 5 Mechanical Engineering Department, College of Engineering at Al Kharj, Prince Sattam Bin Abdulaziz University, Al Kharj 16273, Saudi Arabia; moh.ahmed@psau.edu.sa; 6 School of Engineering, University of Birmingham, Birmingham B15 2TT, UK; k.e.a.essa@bham.ac.uk

**Keywords:** laser powder bed fusion, design of experiments, Ti6Al4V, ANOVA, process parameters

## Abstract

The Ti6Al4V alloy has many advantages, such as being lightweight, formal, and resistant to corrosion. This makes it highly desirable for various applications, especially in the aerospace industry. Laser Powder Bed Fusion (LPBF) is a technique that allows for the production of detailed and unique parts with great flexibility in design. However, there are challenges when it comes to achieving high-quality surfaces and porosity formation in the material, which limits the wider use of LPBF. To tackle these challenges, this study uses statistical techniques called Design of Experiments (DoE) and Analysis of Variance (ANOVA) to investigate and optimise the process parameters of LPBF for making Ti6Al4V components with improved density and surface finish. The parameters examined in this study are laser power, laser scan speed, and hatch space. The optimisation study results show that using specific laser settings, like a laser power of 175 W, a laser scan speed of 1914 mm/s, and a hatch space of 53 µm, produces Ti6Al4V parts with a high relative density of 99.54% and low top and side surface roughness of 2.6 µm and 4.3 µm, respectively. This promising outcome demonstrates the practicality of optimising Ti6Al4V and other metal materials for a wide range of applications, thereby overcoming existing limitations and further expanding the potential of LPBF while minimising inherent process issues.

## 1. Introduction

Additive Manufacturing (AM) has emerged as a transformative technology in the field of manufacturing. It encompasses seven primary technologies. Material Extrusion (ME) involves extruding material through a nozzle, while Vat Photopolymerization (VP) utilises light to selectively cure the liquid resin. Powder Bed Fusion (PBF) employs lasers or electron beams to melt and fuse powder particles, and Material Jetting (MJ) deposits material droplets onto a platform. Binder Jetting (BJ) selectively deposits binding agents onto powdered material, while Directed Energy Deposition (DED) uses thermal energy to melt and deposit material. Lastly, Sheet Lamination (SL) involves joining sheets of material together. These technologies, supported by process optimisation and post-processing, collectively contribute to the diverse landscape of additives [1]. 

Laser Powder Bed Fusion (LPBF) is a widely recognised and popular method in additive manufacturing. It works by using a laser beam to scan a bed of powdered material, melting and fusing the particles together to form a solid object layer by layer. This technique allows for great design flexibility, making it possible to create complex and detailed shapes that were difficult to make using traditional methods. LPBF is particularly useful for the production of small to medium-sized components that require intricate detailing, particularly in biomedical [2], aerospace [3], automotive [4], and chemical and petrochemical applications [5]. One of the primary advantages of LPBF is its capability to produce highly complex geometries and designs that would be impossible to create using conventional manufacturing methods. LPBF can produce fully functional end-use parts directly from digital files, allowing for rapid iterations and improved time-to-market [6]. This makes it an ideal choice for industries that require rapid prototyping as well as the production of highly precise and intricate parts, such as aerospace, medical, and automotive industries. Additionally, LPBF offers exceptional material utilisation, as it minimises waste and allows for the use of metal and its alloys, polymers, and even ceramics [7]. 

The quality of samples produced through LPBF is impacted by several factors, encompassing the powder characteristics and the laser heat input. Powder characteristics, including particle size, shape, distribution, and chemical composition, play a significant role in the consolidation of particles and the occurrence of defects. Meanwhile, the laser heat input holds crucial importance as it governs temperature distribution and melt pool dynamics throughout the additive manufacturing process. Factors such as laser power, scan speed, and spot size contribute to determining the magnitude of the laser heat input [8]. Specifically, higher laser power and slower scan speed are associated with elevated heat input and larger melt pool sizes. These conditions frequently result in enhanced densification and a reduction in defects within the final product. To accurately quantify the laser heat input, the energy density function, symbolised as “Φ”, is utilised. This function defines the energy absorbed by the metal powder per unit volume during the laser scanning process [9]. By utilising the energy density function, the energy input per unit volume can be assessed, enabling the optimisation of process parameters to achieve superior sample quality and consistency [10]. Insufficient laser energy density can lead to the presence of unfused holes in the metal, indicating a lack of complete powder melting. Conversely, excessive laser energy density can cause pores to form as the powder undergoes vaporisation [11].

When compared to other machining methods, the LPBF process has a significant disadvantage in terms of surface quality. This weakness is primarily due to the “stair step” effect created by the process’s layered deposition and fabrication of curves and inclined surfaces, which leads to increased surface roughness. This effect is particularly prominent in parts with complex geometries and can have a significant impact on the end product’s functional performance [12]. Another factor limiting surface quality is the “balling” characteristics that take place during laser melting. The occurrence of the “balling” phenomenon during laser melting can be explained by the fragmentation of the melt pool into small spherical or globular shapes. This phenomenon arises from inadequate adhesion between the molten material and the building platform or substrate, which can be attributed to variations in surface tension caused by discrepancies in thermal properties within the melt pool [13]. This leads to limitations in the LPBF process resolution, making it challenging to achieve sharp geometries. In addition to surface quality, porosity formation is a significant issue that affects part properties such as strength and elastic modulus and can ultimately lead to premature component failure. The formation of porosity in the LPBF process is mainly attributed to the insufficient melting of the metallic powder. As a result, careful control of process parameters, such as laser power, scan speed, hatch spacing, and layer thickness, is necessary to reduce porosity and improve part quality [14,15].

Ti6Al4V, often referred to as the “workhorse” of the titanium industry, is widely recognised as the most extensively utilised titanium alloy. It has earned this distinction due to its remarkable properties, including exceptional mechanical strength, lightweight nature, and outstanding corrosion resistance. These outstanding attributes have positioned Ti6Al4V as a preferred choice for over half of all titanium applications. The distinctive characteristics of Ti6Al4V have established it as a global benchmark across various applications. It finds widespread utilisation in sectors such as aircraft manufacturing, where it is employed for turbines such as engine parts, landing gear components, structural elements, and fasteners [16]. Additionally, Ti6Al4V is extensively used in the automotive industry, particularly in high-performance vehicles. It is used for components that require both strength and lightness, such as exhaust systems, suspension components, and engine parts [17]. Other domains, including biomedical implants [18,19,20], rely on the exceptional properties of Ti6Al4V.

The LPBF process has been widely investigated by researchers studying the properties and characteristics of resulting parts using various approaches. In a study by Song et al. [21], it was found that there is a substantial effect of processing parameters on the roughness, microstructure, microhardness, and densification of Ti6Al4V components manufactured through LPBF. They demonstrated that by controlling the process parameters, it is possible to manufacture Ti6Al4V parts with improved surface finish and eliminated porosity. Yasa et al. [22] utilised laser re-melting (LSR) with a continuous wave laser to improve the surface quality of LPBF-produced Ti6Al4V parts. They reported a surface roughness (Ra) of 15 µm for the LPBF parts without LSR and concluded that by controlling the laser power and scan speed of the LSR process, the surface finish could be significantly improved. At a laser power and laser speed of 256 W and 640 mm/s, respectively, they achieved a roughness of 2.9 µm. Additionally, Attar et al. [23] reported success in producing fully dense titanium composites by optimising LPBF manufacturing parameters using the Taguchi method.

Statistical modelling is a cost-effective and efficient approach for analysing the key parameters that influence the quality and properties of parts. The application of DoE techniques, such as the Response Surface Method (RSM) and statistical analysis using ANOVA, has proven to be highly valuable in investigating the effects of multiple parameters in material processing applications [24]. When multiple parameters and their interactions contribute to the desired outcomes, RSM serves as a valuable statistical tool for quantifying the relation between these parameters and the resulting response surfaces. The ultimate goal is to optimise the process [21]. RSM involves fitting a model to the experimental design data using least squares techniques and then assessing the adequacy of the proposed model through ANOVA tests. Moreover, response surface plots are utilised to visualise the topography of the surface and identify the optimum conditions [25]. Compared to the traditional one-factor-at-a-time approach, optimising processes using RSM is faster and more efficient. Central Composite Design (CCD) is a widely employed RSM approach that plays a vital role in exploring the influence of process parameters on component quality in additive manufacturing processes. CCD incorporates different types of design points, such as two-level factorial points, face points, and centre points, allowing for comprehensive analysis. RSM has successfully been applied in various additive manufacturing endeavours, showcasing its power for assessing the impact of process parameters on component quality [26]. 

However, existing literature on the Laser Powder Bed Fusion (LPBF) of titanium alloys has primarily focused on investigating process parameters and their effects on a specific property like porosity or surface roughness. This approach falls short in providing comprehensive guidance on simultaneously optimising process parameters to achieve an optimum window for both porosity and surface roughness. Consequently, there is a critical need to conduct a multivariate Design of Experiments (DoE) study specifically tailored to Ti6Al4V to address the optimisation of these important properties and their interdependencies, an area that remains largely unexplored. In response to this gap, the primary objective of this work is to develop empirical process models using the DoE approach and statistical regression analysis. By systematically exploring the relationships between process parameters, porosity, and surface roughness, this study aims to develop optimised LPBF parameters for Ti6Al4V components. The specific focus is on enhancing the density and surface quality of Ti6Al4V components fabricated through the LPBF process. To achieve this, a Design of Experiments methodology is employed to assess the influence of laser power, laser speed, and hatch space on the relative density and surface roughness of the produced titanium parts. Subsequently, the LPBF process parameters are optimised to achieve customised parts with tailored properties that are well-suited for aerospace applications, ensuring high density and improved surface quality. Through this research, insights can be obtained regarding the transferability of the processing window across various applications, enabling more versatile and adaptable manufacturing processes. The systematic investigation of process parameters and their effects on Ti6Al4V components will contribute to the existing knowledge base, bridging the gap in understanding and providing guidance for optimising not only Ti6Al4V but also other metal alloys.

## 2. Experimental

### 2.1. Materials

**Figure 1:**
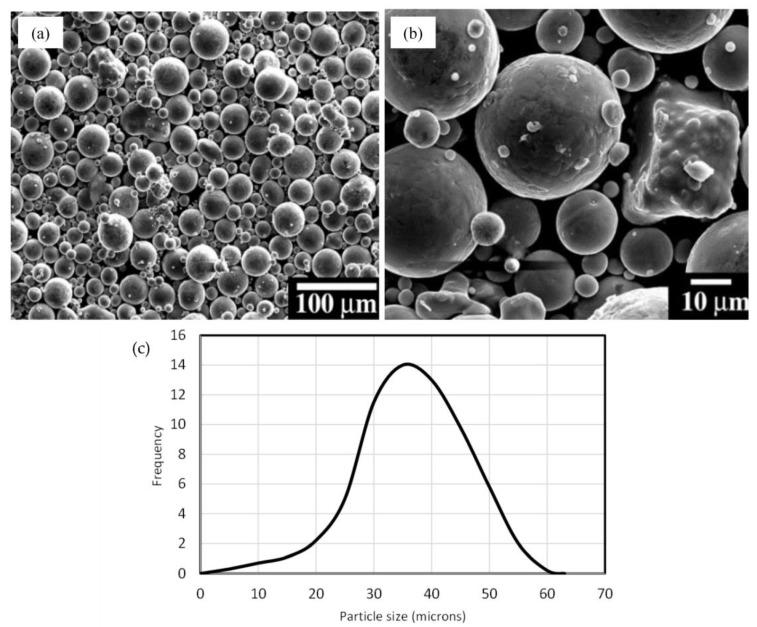
(**a**) An SEM image of Ti6Al4V powder particles, (**b**) a subset of irregular particles, and (**c**) size distribution.

The Ti6Al4V powder utilised in this study was procured from TLS Technik GmbH (Bitterfeld-Wolfen, Germany), and its chemical composition is provided in Table 1. Particle characterisation was conducted using a laser diffraction particle analyser, specifically the Coulter LS230 equipment (Brea, CA, USA), revealing a particle size range of 25–50 µm. The scanning electron microscope (SEM) images, depicted in Figure 1a,b, illustrate predominantly spherical particles with diverse sizes and uniform distribution. However, a few particles exhibit an irregular morphology, as observed in Figure 1b. The size distribution of the powder plays a crucial role in the powder’s flowability in powder bed systems and its melting behaviour during LPBF [8]. The Ti6Al4V powder utilised in this study had a size distribution presented in Figure 1c, with an average particle size of 40 µm. The distribution was somewhat uneven, possibly due to the irregular morphology of some powder particles and their potential agglomeration during measurement. Despite this, the powder exhibited a reasonable flowability and a Hausner’s ratio value suitable for use in LPBF, even with some particles having irregular shapes.

### 2.2. Design of Experiment (DoE)

In this work, RSM was implemented as a technique to optimise the experimental design. By utilising RSM, the researchers were able to identify the relationship between input factors and output responses, thereby facilitating the optimisation of process outcomes. The primary goal is to optimise the response surface, which is influenced by multiple process parameters. The response surface is mathematically represented by a commonly used second-order polynomial equation, as shown in Equation (1). A second-order polynomial equation is often suitable for modelling response surfaces when the relationships between the factors and the response variables are expected to be quadratic [27,28].

(1) $$Y=b_{0}+\sum b_{i}x_{i}+\sum b_{ii}x_{i}^{2}+\sum b_{ij}x_{i}x_{j}\;$$

RSM uses a second-order polynomial equation (regression) represented by model coefficients b_0_, b_i_, b_ii_, and b_ij_, which are determined by the main effects of the process factors and their interactions. The coefficients were determined through the method of least squares. The DoE and parametric optimisation were conducted using Design-Expert Software Version 7.0.0 (Stat-Ease Inc., Minneapolis, MN, USA). The investigation specifically centred on three process parameters: laser power, hatch space, and laser speed. Each factor was varied across five levels (−α, −1, 0, 1, and α). The parameters used for the contour scan were kept consistent with those of the infill scan to ensure uniformity and consistency throughout the process. In addition, there was no specific parametrisation applied to the top surface layer; the parameters were the same as the infill scan. Other parameters related to the machine setups, such as gas flow, are out of the scope of this study.

Table 2 displays specific values for the matrix process parameters in the Concept Laser M2 Cusing LPBF machine. These values were carefully chosen to cover a wide range of operating conditions for effective process optimisation. By using the Central Composite Design methodology, the effects of these parameters on response variables like surface roughness and density were investigated. The selected values represent low, middle, and high levels, allowing for a comprehensive analysis of parameter effects. The choices were based on prior knowledge and preliminary experiments, aiming to capture the nonlinear behaviour of the process and identify optimal settings for achieving desired outcomes. To fabricate the test coupons, 17 parametric combinations employing a response surface DoE were implemented. All the test coupons were cubes of 10 mm × 10 mm × 10 mm dimensions. To examine the impact of the process factors on the properties of interest, Ti6Al4V density and surface roughness were assessed for each sample. Table 2 provides an overview of the critical process variables examined in this study, including their ranges and levels.

### 2.3. Fabrication and Characterisation 

The present study employed a Concept Laser M2 Cusing LPBF system for component fabrication. This system utilises an Nd:YAG laser with a wavelength of 1075 nm, a beam spot size of 50 µm, and a melt pool diameter of approximately 150 µm. The maximum laser output power of the system is 400 W, and it can achieve a maximum laser scanning speed of 7000 mm/s. All manufacturing processes were conducted in an Argon environment with an oxygen percentage of lower than 0.1% [29]. The specimens were built with a vertical Z-increment of 30 µm using an island scanning strategy, a technique where the build is split into multiple square-shaped sections, called islands. The islands in the LPBF process are then scanned in a random manner, and the scan direction is altered by 90° for neighbouring islands. Within each island, the laser follows a raster pattern during scanning. After the completion of scanning all islands at a specific layer, a contour scan is conducted around the build to enhance the surface quality. In the following layer, the islands are moved by 1 mm in both the X and Y. The purpose of employing this island deposition is to minimise developed stresses within the printed parts [30]. To characterise the porosity within the printed Ti6Al4V, the samples were sectioned in the transverse direction at 3 mm from the top of the sample. These sections were then placed in conducting Bakelite resin and polished to obtain a surface finish of 0.05 µm. The polished samples were then examined using a Zeiss Axioskop microscope (Jena, Germany) equipped with an Axioskop 2^®^ image analyser and AxioVision^®^ 4.7.1 software. For each sample, 20 images were captured and analysed using ImageJ 13.0.6 Software to calculate the area fraction of the pores. The relative density of the samples was subsequently computed by subtracting the pore area fraction from 1 [31]. 

The top and side surface roughness of the Ti6Al4V parts was measured using the Surrf.Corder Surface roughness measuring instrument SE 1700 from KosakaLab (Tokyo, Japan). The instrument used for the surface roughness measurements was equipped with a stylus tip that had a radius of 2 µm. The stylus tip is the part of the instrument that physically comes into contact with the surface being measured. A smaller stylus tip radius allows for higher precision in capturing surface details, making it suitable for assessing fine surface roughness features [32,33,34]. 

During the measurements, the instrument’s normal mode was employed, which is a standard setting for surface roughness measurements. The step size used was 313 µm. The step size refers to the distance between consecutive measurement points along the surface. A larger step size enables faster data acquisition but may result in some loss of detail in the surface roughness profile. In our case, the chosen step size of 313 µm was a practical compromise to achieve a balance between measurement speed and resolution. Both the measurement and return speeds were set at 0.1 mms^−1^. Keeping both speeds consistent at 0.1 mms^−1^ ensures uniformity and reliability in the measurement process. 

To ensure accuracy, four measurements were taken for each coupon, with two measurements conducted on the top surface of the sample, and two additional measurements performed on the side surface. The Ra profile was measured along the sample centreline, both horizontally and vertically, at each side. For each measurement, the average surface roughness (Ra) along the centreline was calculated. Then, for each surface (top or side), the mean value of the two measurements was considered as the final Ra value for that surface. 

For visualising the 3D reconstruction of the surface topography, we employed an Alicona Infinite Focus G5 microscope from Graz, Austria. This allowed gaining a comprehensive understanding of the surface quality and its topographical features. Additionally, to examine the phase composition of the Ti6Al4V parts, X-ray diffraction (XRD) analysis was performed using an Inel EQUINOX 3000 instrument from Waltham, MA, USA. The XRD analysis utilised Cu-Kα X-ray radiation with a wavelength of 0.154 nm.

## 3. Results and Discussion

**Figure 2:**
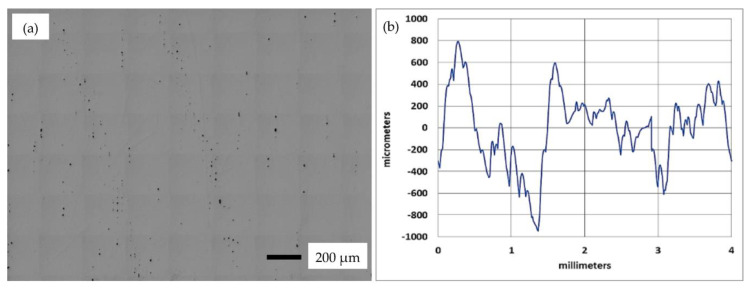
(**a**) A microscope image of a sample cross-section, and (**b**) A typical surface roughness profile of the top surface of sample 3 from the parametric combinations in Table 3.

Table 3 presents the recorded values of relative density, top and side surface roughness, along with the corresponding parametric combinations. Figure 2a displays an optical microscopy image of sample 3, while Figure 2b presents the surface roughness profile of the top surface of the same sample.

### 3.1. ANOVA Results

**Figure 3:**
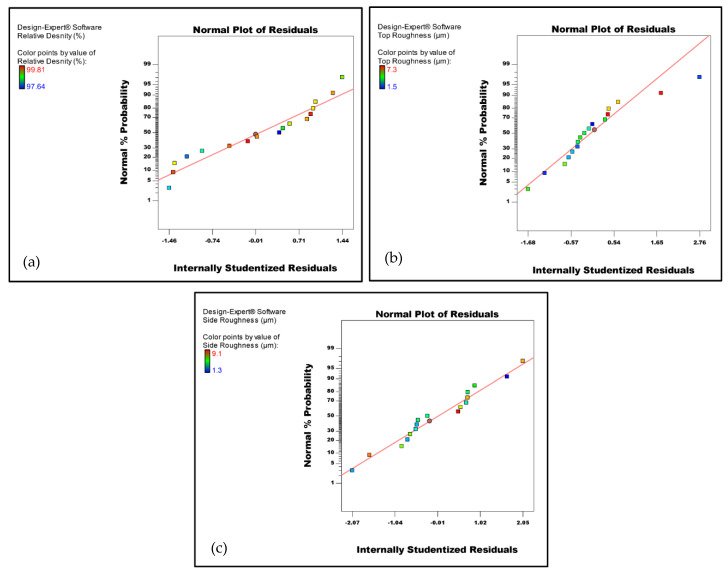
Normal probability plots of the residuals. (**a**) Relative density, (**b**) top roughness, and (**c**) side roughness.

**Figure 4:**
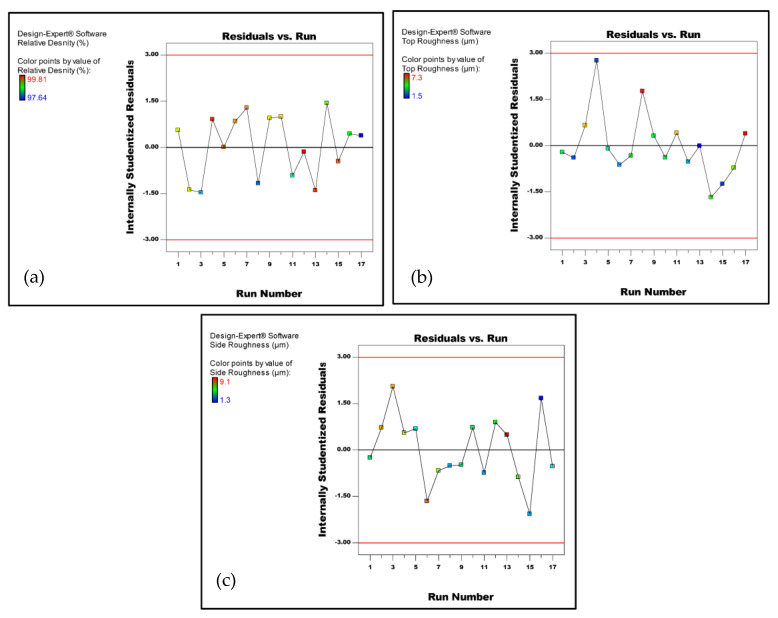
Plots of residuals vs. the run. (**a**) Relative density, (**b**) top roughness, and (**c**) side roughness.

The results presented in Table 3 were used as input into the DoE to perform the ANOVA statistical analysis. Analysis of the fit summary output indicated that the two-factor interaction model was statistically significant for the relative density. On the other hand, the top roughness and side roughness were found to be represented by linear and quadratic functions, respectively, of the process parameters. Therefore, the suggested functions were adopted for the further analysis of these responses. The normal probability of the residuals for the relative density, top and side surface roughness are depicted in Figure 3a–c, respectively. The plots in Figure 3 demonstrated that the residuals were approximately aligned with a straight line, suggesting a normal distribution of the residuals. Additionally, Figure 4a–c displays the residuals-versus-run plots for the three responses, revealing no discernible patterns or anomalies. These findings suggest that the proposed models are suitable, and there is no evidence to suggest a violation of the assumptions regarding the normality and independence of the residuals.

Least Squares Fitting, which is a mathematical procedure for finding the best-fitting curve to a given set of points by minimising the sum of the squares of the offsets of the points from the curve, was applied to analyse the data presented in Table 3. The coefficient of correlation (R^2^) is used to describe the model fit. In this way, the constant coefficients in Equation (1) were determined for all responses considered in the current study. Again, the response surfaces for the relative density, top and side surface roughness were found to fit a two-factor interaction (2FI), linear and quadratic models, respectively, of the laser parameters: laser power (*P*), scan speed (*v*) and hatch space (*h*), with an R^2^ of 0.91, 0.98 and 0.95, respectively, demonstrating a strong correlation. Equations (2)–(4) represent the mathematical expressions for the response surfaces, the relative density, top, and side surface roughness, respectively.

(2) $$\begin{array}{c} Relative\;Density=99.76-8.8\times 10^{-3}\left(P\right)-2.71\times 10^{-4}\left(\nu \right)+7.11\times \\ 10^{-3}\left(h\right)+7.2\times 10^{-6}\left(P\nu \right)+1.2\times 10^{-4}\left(Ph\right)-2.04\times 10^{-5}\left(vh\right) \end{array}$$

(3) $$Top\;Roughness=2.84-0.036\left(P\right)+2\times 10^{-3}\left(\nu \right)+0.04\left(h\right)$$

(4) $$\begin{array}{c} Side\;Roughness=27.58-0.13\left(P\right)-2.09\times 10^{-3}\left(\nu \right)-0.12\left(h\right)-\\ 1.82\times 10^{-5}\left(P\nu \right)+1.09\times 10^{-4}\left(Ph\right)-1.62\times 10^{-5}\left(vh\right)+3.45\times 10^{-4}\left(P^{2}\right)+\\ 1.01\times 10^{-6}\left(v^{2}\right)+4.32\times 10^{-4}\left(h^{2}\right) \end{array}$$

In statistical significance testing, the *p*-value is the probability of obtaining a test statistic at least as extreme as the one that was actually observed, assuming that the null hypothesis is true. The null hypothesis (which assumes that all parameters have no significant effect) is rejected when the *p*-value is less than the predetermined significance level, which is 0.05 (95% confidence level). This means that any factor that has a *p*-value less than 0.05 is considered to be a significant model parameter [35]. Table 4 shows the *p*-value for each parameter and interaction. In this study, the ANOVA results indicated that, within the investigated range of parameters, both the top and side roughness were significantly affected by the laser power, scan speed, and hatch spacing. Additionally, the relative density was suggested to be a function of the three process parameters as well as the interaction between scan speed and hatch spacing.

These results are in agreement with previous research findings. Read et al. [36] suggested that laser power, scan speed, and the interaction between both parameters are significant factors influencing porosity development in LPBF of AlSi10Mg alloy. Similarly, Song et al. [21] reached similar conclusions regarding the Ti6Al4V alloy. The role played by the laser parameters in controlling the surface finish of SLM parts was also reported by several literature sources [13,21,37].

#### 3.1.1. Relative Density Analysis

**Figure 5:**
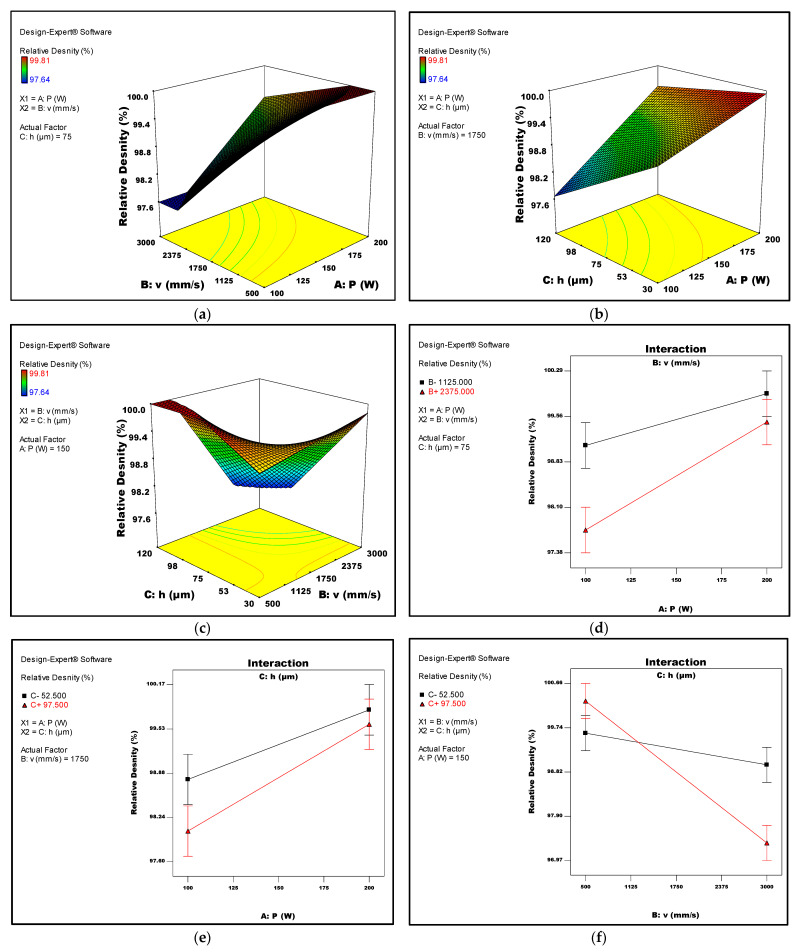
(**a**–**c**) Response surface of the relative density against LPBF process parameters (**a**) laser power and scan speed, (**b**) laser power and hatch space, and (**c**) scan speed and hatch space. (**d**–**f**) Interaction plots of the relative density against LPBF process parameters (**d**) laser power and scan speed, (**e**) laser power and hatch space, and (**f**) scan speed and hatch space.

Figure 5a–c present 3D surface plots that visualise the relation between the relative Ti6Al4V density and the laser power, scan speed, and hatch spacing, respectively. Corresponding interaction plots between the laser parameters and the relative density are presented in Figure 5d–f. These plots revealed that increasing the laser power or reducing the value of the scan speed and hatch space can lead to an improvement in the relative density. Additionally, Figure 5c,f highlights the significant interaction between hatch space and scan speed. It is worth noting that maintaining an adequate energy density is crucial to prevent incomplete melting. Insufficient consolidation and a smaller melt pool can occur when scan speed and hatch space are increased or when laser power is decreased. As a consequence, voids can become trapped between the powder particles under the solidified hatch lines, ultimately diminishing the overall density of the LPBF part.

#### 3.1.2. Surface Roughness Analysis 

**Figure 6:**
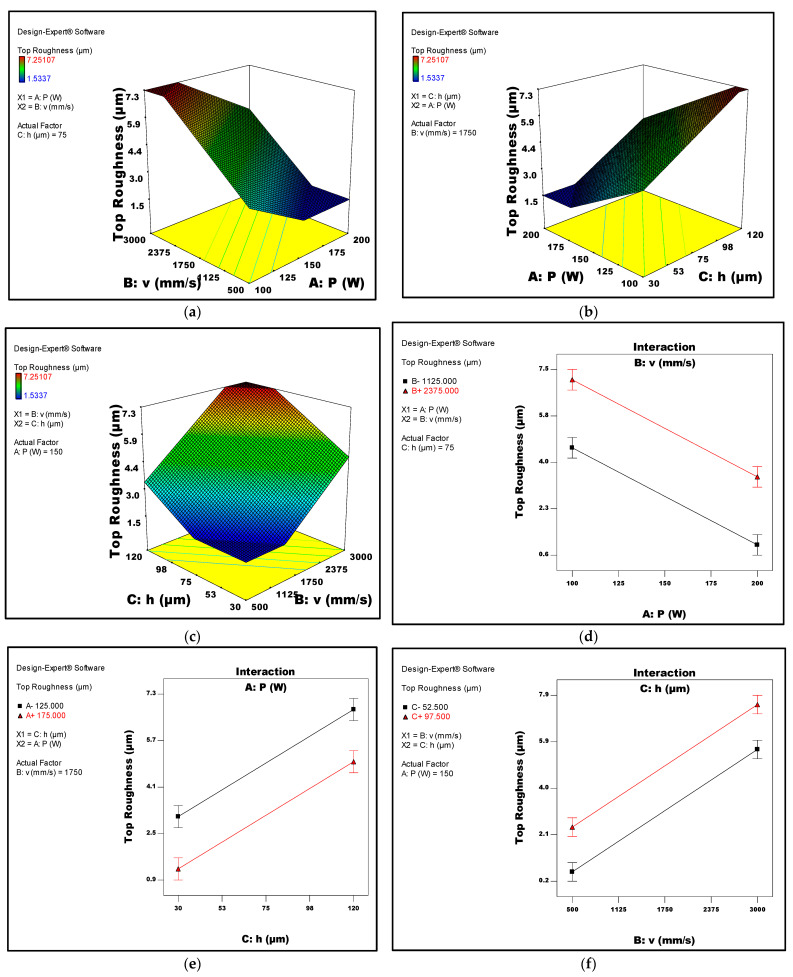
(**a**–**c**) Response surface plots depicting the top surface roughness in relation to the LPBF process parameters (**a**) laser power and laser speed, (**b**) laser power and hatch space, and (**c**) scan speed and hatch space. (**d**–**f**) Interaction plots of the top roughness against LPBF process parameters, (**d**) laser power and scan speed, (**e**) laser power and hatch space, and (**f**) scan speed and hatch space.

**Figure 7:**
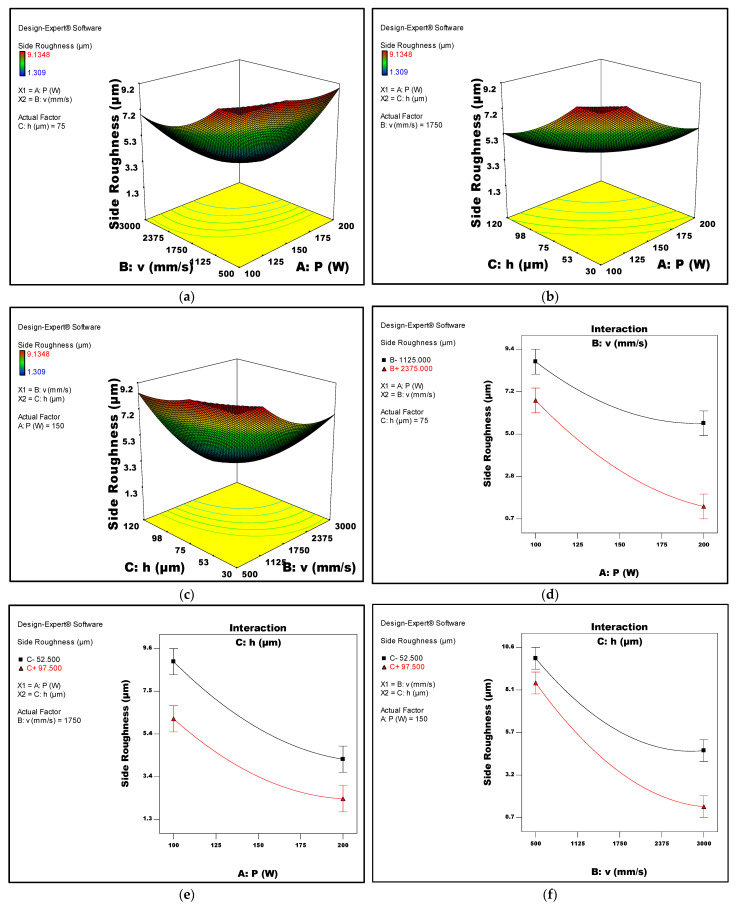
(**a**–**c**) Response surface plots depicting the side surface roughness in relation to the LPBF process parameters, (**a**) laser power and laser speed, (**b**) laser power and hatch space, and (**c**) scan speed and hatch space. (**d**–**f**) Interaction plots of the side roughness against LPBF process parameters, (**d**) laser power and scan speed, (**e**) laser power and hatch space, and (**f**) scan speed and hatch space.

Figure 6a–c depicts 3D surface plots illustrating the relationship between the top surface roughness of LPBF samples and the respective process parameters of laser power, scan speed, and hatch space, respectively. Interaction graphs between the LPBF parameters and the top roughness are given in Figure 6d–f. Corresponding surface plots and interaction graphs that describe the relationship between the process parameters and side surface roughness are given in Figure 7a–f. It was shown that both roughness types were suggested to decrease with increasing the laser power. However, increasing the scan speed and/or hatch spacing was found to increase the top roughness while significantly reducing the side roughness.

It could be suggested that decreasing the hatch spacing generally increases the overlapping of laser spots which decreases the variation in the surface profile and leads to an enhancement in the surface finish of the top surface of the LPBF part. The smoothness of the top surface could be further improved by using low scan speeds, which encourages the relaxation of the melt pool as there would be sufficient time for the forces by gravity and surface curvature to neutralise the shear forces resulting from the temperature gradient inside the molten pool. This allows the melt pool to flatten before its complete solidification and eliminates the top surface’s roughness. However, reducing the hatch spacing and scan speed might widen the melt pool and increase the inhomogeneity of the thermal properties and surface tension across the pool. In order to decrease the variation in the surface tension, the melt pool detaches into smaller spheres, which is called the “balling effect”, which results in a poor finish of the side surfaces of the SLM component. These findings align with a previous study conducted by Mumtaz et al. during their study of the top and side surface side roughness LPBF components from Inconel 625 super alloy [13]. 

ANOVA analysis also revealed that increasing the laser power could significantly reduce the roughness of both the top and side surface of the LPBF parts. This was demonstrated by the clear trends shown in Figure 6 and Figure 7: as the laser power increases, the top and side surface roughness of LPBF parts decrease significantly. This reduction can be attributed to the improved consolidation of metal powder which improved the interlayer connection and eliminated the presence of spatter particles on the surface. As a result, a superior top surface finish is achieved [38,39]. Furthermore, the higher laser power also enhanced the wettability of the sample melt pool, thereby reducing disparities in surface tension. This improvement in wettability plays a crucial role in minimising the occurrence of the balling phenomenon, which is known to have a detrimental effect on side surface roughness [40]. Wang et al. [39], where the effect of a range of SLM laser powers (100–200 W) and scan speeds (600–1600 mm/s) was investigated on the roughness of Ti6Al4V parts, it was shown that increasing the laser power to the range from 180 to 200 W caused a substantial decrease of the roughness of both the upper and side surfaces of the components. 

It was also suggested that the top roughness is affected by the combination of scanning speed and laser power. Higher laser powers, along with smaller scan speeds, would cause the laser to melt more metal powder, improving the molten metal liquid wettability, inhibiting the appearance of spheroidisation, and in turn, results in a more regular and continuous melting channel, which decreases the top surface roughness. This is clearly shown in Figure 6d, where the least top roughness was suggested to result in high laser power and low scan speed.

It should be noted that the conclusions regarding the porosity and the surface roughness are specifically applicable within the range of process parameters investigated in this study. It is important to consider that beyond this range, additional factors such as melt pool turbulence or evaporation can also have an influence. These factors could contribute to the formation of porosity or potentially affect the melt pool stability, thereby resulting in surface roughness (Ra) increase, particularly at the top surface of the fabricated part. 

### 3.2. Process Optimisation

**Figure 8:**
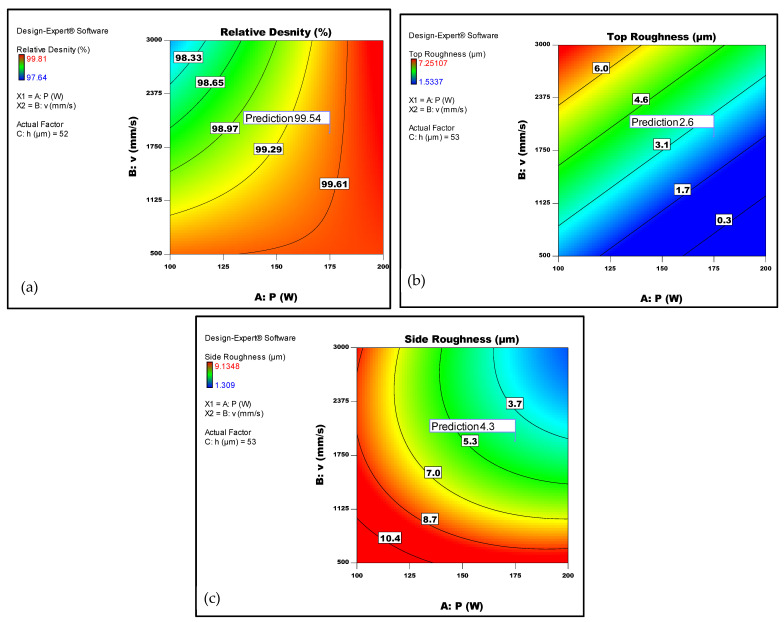
Predicted optimum laser parameters for (**a**) maximum relative density, (**b**) minimum top roughness, and (**c**) minimum side roughness.

The primary aim of this research was to optimise the processing parameters of LPBF in order to achieve the maximum density and the best surface finish on both the top and side surfaces of fabricated parts which were considered to have the same weight. To accomplish this, design-expert software was employed to conduct the optimisation analysis. The genetic algorithm was utilised to estimate the optimal process parameters based on the defined objective function. By simultaneously solving Equations (2)–(4), which describe the relationship between relative density and top and side surface roughness, respectively, with respect to the LPBF laser parameters, the desired outcomes were obtained. The results generated by the design-expert software are depicted in Figure 8, which illustrates a contour plot representing the optimisation function. This plot showcases the range of scan speeds and laser powers required to achieve maximum density and minimum roughness.

According to the model, the optimised setting for the process factors was determined to be 175 W for laser power, 1914 mm/s for scan speed, and 53 µm for hatch space. This configuration corresponds to an energy density of 58 J/mm^3^. With these settings, the predicted relative density, top and side surface roughness of an LPBF Ti6Al4V part were estimated to be 99.54% and 2.6 µm and 4.3 µm, respectively. 

In a previous study carried out by Pal et al. [41], successful manufacturing of a Ti6Al4V part using selective laser melting was reported, with the following parameter values: 75 Watt laser power, 600 mm/s laser scan speed, 77 µm hatch space, and 25 µm layer thickness, resulting in an energy density of 65 J/mm^3^. With these parameters, the achieved relative density was 99.45%. In addition, the roughness of the vertical surfaces at these values of process parameters was found to be around 5 µm [42]. In the current study, the energy density associated with the optimised process parameters was 58 J/mm^3^ which is close to that reported by Pal et al. The estimated values of the relative densities were almost the same, while those for the side roughness were also too close. 

In another study by Palmeri et al. [43], the authors demonstrated that the use of 350 Watt laser power, 1400 mm/s scan speed, 120 μm scan space, and 30 μm layer, corresponding to a volumetric energy density of 69 J/mm^3^, was sufficient to obtain nearly fully dense Ti6Al4V samples. The energy density in this study was slightly higher than that predicted in the current study, which might cause the relative density to reach about 100%. The minor differences between the energy density associated with the optimised process parameters in the current study and those suggested by Pal and Palmeri might be due to the fact that in the current study, the process parameters were optimised to achieve not only the highest relative density but also the lowest top and side surface roughness. 

### 3.3. Model Validation

**Figure 9:**
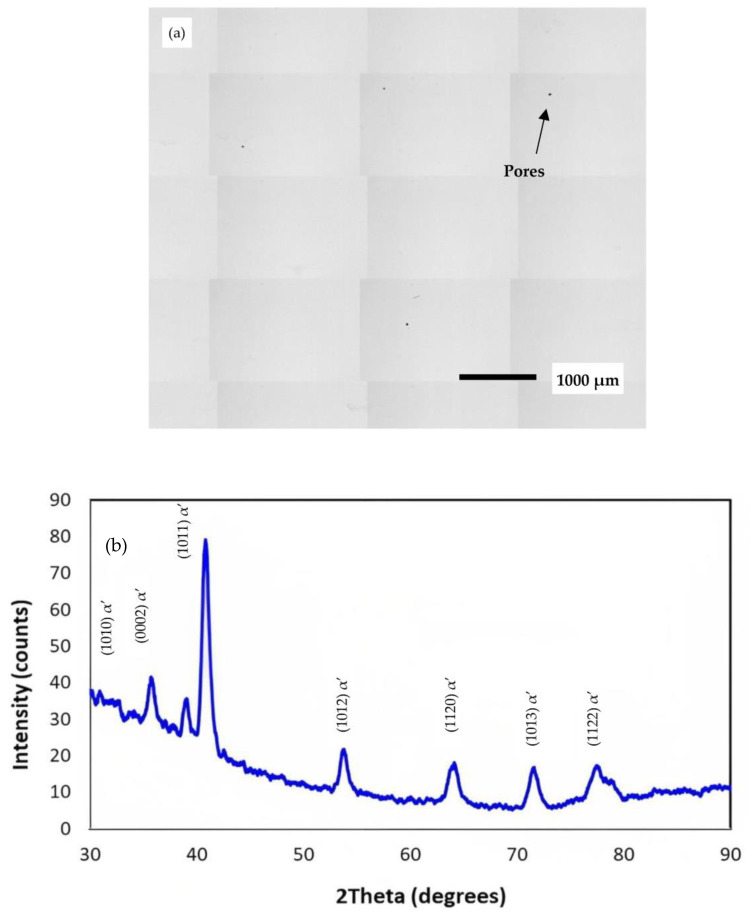
(**a**) A microscope image showing the cross-section, (**b**) XRD measurement of the as-fabricated Ti6Al4V sample. All samples were produced using the optimised parameters.

To validate the results obtained from the process optimisation, five LPBF cubes were fabricated with the same dimensions as the test coupons used in the DoE investigation. The recommended values provided by the model for achieving optimal properties were implemented, which included a laser power of 175 W, scan speed of 1914 mm/s, and hatch space of 53 µm. The layer thickness was kept constant at 30 µm. The five samples were characterised the same way as the 17 parametric DoE samples. Upon analysing the results, the average measured values of the porosity and top and side surface roughness were determined to be 99.65%, 2.8 μm, and 4.29 μm, respectively. To further emphasise the quality of the fabricated samples. The consistency between these measured values and the predicted outcomes based on the optimised process parameters reaffirms the effectiveness of the suggested parameters in achieving the optimum material properties. Figure 9a shows the cross-section optical microscopy image of the sample built using the optimal conditions showing a few small pores. The microstructural characterisation of the as-fabricated Ti-6Al-4V sample was performed using X-ray diffraction (XRD), as shown in Figure 9b. The XRD analysis revealed the presence of a martensitic needle-like α’ structure as the dominant phase in the sample. No peaks corresponding to the β-Ti phase were observed. The development of the α’ structure in the as-fabricated sample is consistent with findings reported in several literature sources, including references [32]. This agreement indicates that the observed microstructure is in line with established research in the field. Furthermore, the intensity of the α-Ti (1011) peak in the as-fabricated condition was found to be significantly higher compared to the other intensities. This suggests that the α-Ti (1011) crystallographic plane is preferentially oriented or has a higher volume fraction within the microstructure.

### 3.4. Relative Density and the Energy Density

**Figure 10:**
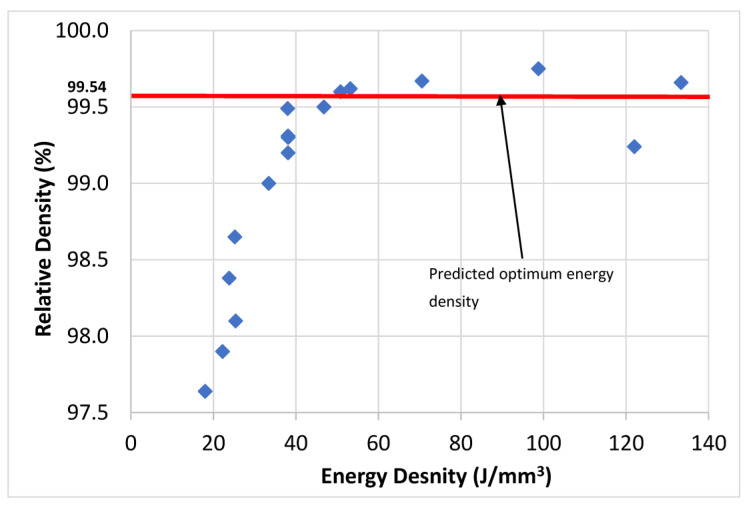
Relative density variation versus energy density.

Figure 10 provides the relation between Ti6Al4V relative density and laser volumetric energy density, utilising the data presented in Table 3. The red line on the graph represents the predicted optimal level of porosity based on the optimal process parameters. The figure demonstrates a consistent improvement in relative density as the energy density increases, exceeding 99.5% at a range approximately between 50 and 100 J/mm^3^. This improvement can be attributed to two key factors. Firstly, the increased energy density promotes better consolidation of the metal particles, leading to a more compact and solid structure. Secondly, the higher energy density enhances the fluidity of the metal within the molten pool, enabling it to effectively fill the gaps between the powder particles. Consequently, this eliminates the formation of porosity and enhances the overall relative density of the LPBF samples. However, it is important to note that beyond the optimum energy density level, which corresponds to the peak relative density, the graph demonstrates a scattering of relative density values that exceed the optimal porosity level, reaching as high as 140 J/mm^3^. This scattering can be attributed to the excessive input of laser irradiation energy, which causes substantial vaporisation of the aluminium from the alloy and a subsequent formation of keyholes within the material. These keyholes are voids or cavities that negatively impact the material density, resulting in a decrease in the relative density of Ti6Al4V samples. It is worth mentioning that previous studies have reported the existence of a threshold energy density specific to different alloy systems. For Al-Si-Mg alloys, this threshold energy density was typically observed between 60 and 75 J/mm^3^ [33,44]. In the case of Al0.5CoCrFeNi high entropy alloys, the threshold energy density was approximately 150 J/mm^3^. This threshold signifies the point, or range, at which the material density reaches its maximum value, beyond which further increases in energy density might lead to a decline in relative density. This graph defines the relation between energy density and the sample’s relative density in LPBF processes. They emphasise the importance of carefully selecting and controlling the energy density to achieve optimal material density and avoid excessive vaporisation and porosity formation [45].

## 4. Conclusions

This study addressed the critical need for comprehensive optimisation of Laser Powder Bed Fusion (LPBF) process parameters in titanium alloy (Ti6Al4V) fabrication. Prior research had focused on individual property investigations, such as porosity or surface roughness, without considering their interdependencies. To fill this gap, a multivariate Design of Experiments (DoE) approach was utilised to develop empirical process models and explore the relationships between process parameters, relative density, and top and side surface roughness. The ANOVA analysis demonstrated the significant effect of the studied process parameters on the relative density and surface roughness of test coupons. Increasing the laser power positively affected the relative density while simultaneously reducing top and side surface roughness. On the other hand, scan speed and hatch space exhibited a reverse effect, decreasing both the relative density and side roughness while might result in rising the top surface roughness. This study emphasised the careful optimisation of these parameters to ensure desired material density during LPBF. An optimisation study led to the identification of optimal processing conditions for Ti6Al4V components. By using a laser power of 175 W, a scan speed of 1914 mm/s, and a hatch space of 53 µm, a remarkable relative density of 99.54%, a top roughness of 2.6 µm, and a side roughness of 4.3 µm were achieved. The findings of this research not only contribute to improving the performance of Ti6Al4V components but also hold potential applications for various alloys and materials, enabling enhanced quality and functionality in a wide range of additively manufactured components. By providing valuable insights into the interplay between process parameters and material properties, this study opens new directions for further advancements in the field of LPBF and additive manufacturing as a whole.

## Figures and Tables

**Table 1:** Ti6Al4V Chemical composition (wt. %) as supplied by TLS Technik GmbH (Bitterfeld-Wolfen, Germany).

Element	Al	V	Fe	C	O	N	H	Ti
wt. %	5.9	3.98	0.158	0.006	0.160	0.004	0.002	Bal.

**Table 2:** The matrix process parameters.

Parameter	Levels				
	−α	−1	0	1	α
Laser Power (W)	100	125	150	175	200
Laser Scan Speed (mm/s)	500	1125	1750	2375	3000
Hatch Space (µm)	30	52.5	75	97.5	120

**Table 3:** Matrix building parameters, surface roughness (Ra) and relative density.

Run	Laser Power (W)	Scan Speed (mm/s)	Hatch Space (µm)	Energy Density (J/mm^3^)	Relative Density (SD) %	Top Roughness, (SD) µm	Side Roughness, (SD) µm
1	150	1750	75	38	99.20 (0.07)	4.0 (0.23)	4.2 (0.26)
2	150	1750	30	95	99.24 (0.05)	2.1 (0.17)	7.7 (0.34)
3	100	1750	75	25	98.10 (0.07)	6.1 (0.22)	7.8 (0.42)
4	175	1125	52.5	99	99.75 (0.06)	2.0 (0.17)	6.9 (0.38)
5	175	2375	52.5	47	99.50 (0.04)	3.5 (0.25)	3.7 (0.19)
6	125	1125	52.5	71	99.46 (0.08)	2.5 (0.18)	8.2 (0.41)
7	125	1125	97.5	38	99.49 (0.06)	4.5 (0.22)	6.3 (0.24)
8	150	3000	75	22	97.90 (0.07)	7.2 (0.32)	2.7 (0.14)
9	150	1750	75	38	99.30 (0.08)	4.2 (0.21)	4.2 (0.25)
10	150	1750	75	38	99.31 (0.04)	3.9 (0.18)	4.5 (0.28)
11	150	1750	120	24	98.38 (0.05)	6.0 (0.23)	2.7 (0.15)
12	175	1125	97.5	53	99.81 (0.04)	2.6 (0.14)	5.3 (0.28)
13	150	500	75	133	99.66 (0.03)	1.5 (0.22)	9.1 (0.48)
14	125	2375	52.5	33	99.00 (0.05)	4.7 (0.24)	6.3 (0.32)
15	200	1750	75	51	99.60 (0.06)	1.8 (0.13)	2.6 (0.18)
16	175	2375	97.5	25	98.65 (0.07)	5.1 (0.27)	1.3 (0.25)
17	125	2375	97.5	18	97.64 (0.05)	7.3 (0.26)	3.3 (0.35)

**Table 4:** ANOVA *p*-values for each of the parameters and parameter interactions for the relative density, top and side roughness.

Model Parameter	*p*-Value		
	Relative Density	Top Roughness	Side Roughness
*P*	**0.0006**	**<0.0001**	**<0.0001**
*V*	**<0.0001**	**<0.0001**	**<0.0001**
*h*	**0.0043**	**<0.0001**	**<0.0001**
*PV*	0.2515	N.A.	0.0626
*Ph*	0.4820	N.A.	0.4337
*vh*	**0.0110**	N.A.	0.1197
*P* ^2^	N.A.	N.A.	**0.0351**
*v* ^2^	N.A.	N.A.	**0.0020**
*h* ^2^	N.A.	N.A.	**0.0333**

Bold values indicate statistically significant process parameters (*p*-value < 0.05).

## Data Availability

All data presented in this paper that support the findings of this study are included within this paper. Additional data are available from the corresponding author upon reasonable request.
